# Effects of *Leymus chinensis* hay and alfalfa hay on growth performance, rumen microbiota, and untargeted metabolomics of meat in lambs

**DOI:** 10.3389/fvets.2023.1256903

**Published:** 2023-11-16

**Authors:** Hanning Wang, Lingbo Meng, Lan Mi

**Affiliations:** State Key Laboratory of Reproductive Regulation and Breeding of Grassland Livestock, Key Laboratory of Forage and Endemic Crop Biotechnology, Ministry of Education, School of Life Sciences, Inner Mongolia University, Hohhot, China

**Keywords:** alfalfa hay, growth performance, lamb, *Leymus chinensis* hay, metabolomics, rumen bacteria

## Abstract

**Objective:**

The objective of this study was to compare the effects of *Leymus chinensis* hay and alfalfa hay as the roughage on the rumen bacterial and the meat metabolomics in lambs.

**Methods:**

Fourteen male lambs were randomly assigned to two dietary treatments (one group was fed with concentrate and *Leymus chinensis* hay; another was fed with concentrate and alfalfa hay) with seven replicates per treatment. The feeding experiment lasted for 60 days. Lambs were slaughtered at the end of the feeding experiment. Growth performance, carcass performance, and weights of various viscera were determined. The longissimus dorsi and rumen contents were collected for untargeted metabolomics and 16S rDNA amplicon sequencing analysis, respectively.

**Results:**

The lambs fed with alfalfa hay showed a significantly increased in average daily gain, carcass weight, dressing percentage, loin-eye area, and kidney weight. Feeding *Leymus chinensis* hay and alfalfa hay diets resulted in different meat metabolite deposition and rumen bacterial communities in the lambs. The relative abundance of phyla *Fibrobacteres*, *Bacteroidetes*, and *Spirochaetes* were greater in the *Leymus Chinensis* hay group, while, the relative abundance of *Firmicutes*, *Proteobacteria*, *Fusobacteria*, and *Verrucomicrobia* were greater in the alfalfa hay group. Based on untargeted metabolomics, the main altered metabolic pathways included alanine, aspartate and glutamate metabolism, D-glutamine and D-glutamate metabolism, phenylalanine metabolism, nitrogen metabolism, and tyrosine metabolism. Several bacteria genera including *BF31*, *Alistipes*, *Faecalibacterium*, *Eggerthella*, and *Anaeroplasma* were significantly correlated with growth performance and meat metabolites.

**Conclusion:**

Alfalfa hay improved growth performance and carcass characteristics in lambs. *Leymus chinensis* hay and alfalfa hay caused different meat metabolite deposition by modifying the rumen bacterial community. These findings will be beneficial to future forage utilization for sheep growth, carcass performance, and meat quality improvement.

## 1. Introduction

Mutton is one of the most widely consumed meats around the world due to its high protein and low cholesterol (1). With the growing requirement for high-quality meat, meat quality has induced more and more attention. Improving animal diets is one of the most effective ways to ameliorate animal growth performance, carcass traits, and meat quality (2, 3). Roughage is a necessary nutrient source for ruminants. In particular, types and quality of forage are key factors affecting ruminant productivity, carcass composition, rumen microbiota, and quality of meat nutrition such as amino acids, fatty acids, and mineral elements (4, 5).

*Leymus chinensis*, a perennial species of Gramineae, is widely distributed in the Eurasian Steppe including the eastern Inner Mongolian Plateau and the Songnen Plain in China (6). *Leymus chinensis* has been one of the main forages due to its high yield, appropriate nutritional values, and palatability (7). It has been reported that adding *Leymus chinensis* hay in the diet to replace part of the corn silage and alfalfa hay facilitated the improvement of milk yield, milk fat and protein yield, and milk fat concentration (8). Enhancing the ratio of *Leymus chinensis* silage decreased dry matter and neutral detergent fiber degradability but promoted crude protein degradability in the combinations of *Leymus chinensis* silage and corn silage in beef cattle (7). Moreover, the *Leymus chinensis* hay diet increased the C15:0 fatty acid contents in lamb meat compared to the mixed forage diet (5). As the average daily gain (ADG) of ewes was higher in the *Leymus chinensis* hay treatment group, *Leymus chinensis* hay was of better quality for ewes than *Vigna radiata* stalk (9).

Alfalfa hay is famous for its high quality and widely used as an important dietary roughage for ruminants. Feeding with alfalfa hay increased the growth performance in lambs, such as the ADG, compared with wheat straw diets (10). Feeding lactating ewes with alfalfa hay promoted milk production compared with the wheat straw diets (10). A forage diet mixture of alfalfa hay and maize stover in a ratio of 60:40 optimized the growth and carcass traits of lambs (11). Additionally, alfalfa hay as an ingredient to supplement the low-energy diet increased omega-3 fatty acids and lowered the omega-6: omega-3 ratio in lamb meat (12), indicating that alfalfa affects the meat metabolites and nutritional quality.

The rumen is a complex ecosystem containing functional microbiota responsible for the rumen fermentation and an important part in producing nutrient substances and calories in ruminants (13, 14). The ruminal bacteria community is closely correlated with diets (15). Interestingly, altering the diet has a cascading effect on the rumen microbiota, which affects animal growth performance and meat quality (15). Previous reports have revealed that various forages have a great effect on rumen and fecal microbiota composition (16–18). For example, lambs fed with alfalfa had a higher relative abundance of *Akkermansia* and *Asteroleplasma* than the mix of purple prairie clover and alfalfa treatment group (18). The alfalfa hay diet elevated the proportion of *Prevotella* and *Selenomonas* compared with the cornstalk diet, while cornstalk feeding increased the proportion of *Thermoactimoyces*, *Bacillus*, *Papillibacter*, *Anaerotruncus*, and *Streptomyces* compared with the *Leymus chinensis* hay or alfalfa hay feeding in dairy cows (19). It has also been revealed associations between the rumen bacterial community with metabolite deposition. For instance, *Bacteroidales_UCG-001_norank* was negatively related to fatty acids including C18:2 and C20:4 in the longissimus dorsi (LD) of sheep (20). Amino acids including isoleucine and glycine were positively correlated with *Anaeroplasma* and negatively associated with *Parabacteroides* and *Alloprevotella* (21). *Moryella* was positively associated with fatty acids such as C16:0 and C18:1n9c, and negatively related to C20:4 n6, C20:3 n6, and C20:5 in lamb lumborum muscle (22). *Moryella* also exhibited a positive relationship with meat metabolites including L-carnosine, N-acetyl-L-histidine, and negatively related to N-acetylaspartylglutamate, L-carnitine, L-citrulline, and Pro-Glu (22). Therefore, it is important to characterize the relationship between rumen bacteria and meat metabolites.

To our knowledge, the comparison of growth performance, carcass characteristics, and meat metabolites of sheep fed with *Leymus chinensis* hay and alfalfa hay remains poorly defined. The alterations in the rumen bacterial community and interactions between bacteria and metabolites are also lacking. The lack of in-depth evaluation may affect the application of forages in ruminant farming. Therefore, this study focused on investigating the effects of *Leymus chinensis* hay and alfalfa hay on growth and carcass traits, meat metabolite deposition, and rumen bacterial community in lambs. This study will facilitate assessing growth and carcass performance, and meat quality of sheep fed with forages different forage types, and provide an important reference for improving ruminant growth and carcass performance through manipulating diets in the future.

## 2. Materials and methods

### 2.1. Animals and experimental design

The animal protocol in this study was approved by the Animal Care and Use Committee of Inner Mongolia University (Approval No. IMU-sheep-2020-041).

Healthy male East Friesian × Small-tail Han lambs were purchased from Inner Mongolia Lark Biotechnology Co., Ltd. Fourteen lambs with an average age of 60 days and with body weight of about 22.03 ± 1.08 kg were selected. The feeding trials were conducted at the Inner Mongolia University Meat Sheep Nutritional Base, where each lamb was housed individually in the same pens. Fourteen lambs were randomly allocated to two dietary treatment groups: one group was feed with concentrate and *Leymus chinensis* hay (Lc group); another was fed with concentrate and alfalfa (*Medicago sativa*) hay (Ms group) with free access to water. The Lc group consumed about 325 g of concentrate and 803 g of (*Leymus chinensis* hay each sheep per day, while the Ms group consumed about 325 g of concentrate and 925 g of alfalfa hay each sheep per day. Ingredient and chemical compositions of diets are shown in Supplementary Table S1. Before the experiment, lambs were acclimatized to the environment for about 1 week. The experiment lasted for 60 days. At the end of the experiment, lambs were selected for slaughter after fasting for about 12 h, and the LD and rumen contents samples were collected. Muscle samples were collected using a disposable scalpel at the same location in the LD of each lamb into 50 mL RNAase-free centrifuge tubes and stored at −80°C for metabolite analysis. After the rumen was opened, the rumen contents were filtered using 4 layers of sterile gauze, and the rumen contents were collected in a 50 mL RNAase-free centrifuge tube and stored at −80°C for 16S rDNA amplicon analysis.

### 2.2. Chemical analysis of roughage (*Leymus chinensis* hay and alfalfa hay)

Dry matter and crushed ash of *Leymus chinensis* hay and alfalfa hay samples were determined according to Ran et al. (23). Crude protein, acid detergent fiber, neutral detergent fiber, and crude fat in the *Leymus chinensis* hay and alfalfa hay samples were determined using an Automatic Kjeldahl Protein/Nitrogen Analyzer (K1160, Hanon Advanced Technology Group, China), Automatic Fiber Analyser (F2000, Hanon Advanced Technology Group, China) and Automatic Soxhlet Extractor (SOX606, Hanon Advanced Technology Group, China) respectively with reference to Shi et al. (24). The content of mineral elements was determined with using microwave (REVO, LabTech, China) and inductively coupled plasma-optical emission spectrometer (PQ 9000, analytikjena, German) (25).

### 2.3. Determination of growth and carcass performance and organ index

Body weight and carcass weight were measured following overnight fasting and evisceration, respectively. Loin-eye area between the 12th and 13th ribs was traced on sulphate papers and calculated (26). Dressing percentage = carcass weight/body weight. Organ index = organ weight/body weight (27). Total weight = final body weight (FBW) − initial body weight (IBW). Average daily gain (ADG) = total weight gain/total days. Feed to gain ratio (F/G) = average daily feed intake (ADFI)/ADG.

### 2.4. 16S rDNA amplicon sequencing and bioinformatics analysis

The rumen contents were collected and stored at −80°C. Microbial DNA was extracted using a TIANGEN kit. The V3–V4 region of the bacterial 16S rDNA gene was amplified by PCR (95°C for 3 min, followed by 30 cycles at 95°C for 30 s, 50°C for 30 s, 72°C for 45 s, and an extension at 72°C for 10 min) using the F3 (ACTCCTACGGGAGGCAGCAG) and R4 (GGACTACHVGGGTWTCTAAT) primer pair (28). DNA Library Prep Kit for Illumina following manufacturer’s recommendations and index codes were added. High throughput sequencing was performed utilizing the Illumina MiSeq PE300 platform to detect the 16S rDNA amplicons according to standard protocols.

After sequencing, paired-end reads from sequencing were merged by FLASH, and low-quality reads were filtered by Trimmomatic. UPARSE was used to align operational taxonomic units (OTUs) at 97% identity. Taxonomy was assigned to OTUs by searching against the Greengenes database version 13.8. α- and β- diversities were calculated using QIIME2. The biomarkers with statistical differences were screened by Linear discriminant Effect Size (LEfSe) analysis, and the screening criteria were LDA threshold ≥4.0 and *p*-value <0.05. The redundancy analysis (RDA) and Spearman’s rank correlation analysis were performed using the R packages: vegan and pheatmap, respectively.

### 2.5. Untargeted metabolomics and bioinformatics analysis

Untargeted metabolomics analysis was referenced to the previous method (29, 30). Briefly, 200 mg LD samples were mixed with 2-chlorophenylalanine (4 ppm) methanol (−20°C) and ground by a high-throughput tissue grinder for 90 s at 60 Hz. The samples were centrifuged at 4°C for 10 min at 12,000 rpm, and the supernatant was filtered through 0.22 μm membrane to obtain the prepared samples for LC-MS. Twenty microlitre from each sample were taken to the quality control samples, and the rest of the samples were used for LC-MS detection.

LC-MS detection was referenced against the previous method (31). Chromatographic separation was accomplished in a Thermo Ultimate 3000 system equipped with an ACQUITY UPLC^®^ HSS T3 column maintained at 40°C. The temperature of the autosampler was 8°C. Gradient elution of analytes was carried out with 0.1% formic acid in water (C) and 0.1% formic acid in acetonitrile (D) or 5 mM ammonium formate in water (A) and acetonitrile (B) at a flow rate of 0.25 mL/min. Injection of 2 μL of each sample was performed after equilibration. An increasing linear gradient of solvent B (v/v) was used as follows: 0–1 min, 2% B/D; 1–9 min, 2%–50% B/D; 9–12 min, 50%–98% B/D; 12–13.5 min, 98% B/D; 13.5–14 min, 98% B/D; 14–20 min, 2% D-positive model (14–17 min, 2% B-negative model) (31).

The ESI-MSn experiments were executed on the Thermo Q Exactive Focus mass spectrometer with the spray voltage of 3.8 kV and −2.5 kV in positive and negative modes, respectively. Sheath gas and auxiliary gas were set at 30 and 10 arbitrary units, respectively. The capillary column temperature was 325°C. The analyzer scanned over a mass range of *m*/*z* 81–1,000 for a full scan at a mass resolution of 70,000. Data-dependent acquisition (DDA) MS/MS experiments were performed with an HCD scan. The normalized collision energy was 30 eV. Raw data were converted to the mzXML format by Proteowizard (v3.0.8789), and then processed by the MetaboAnalystR package (32).

### 2.6. Statistical analyses

GraphPad Prism was used for statistical analysis. The results of growth performance, carcass performance, and organ index are presented as means ± standard error (SE). All data were considered statistically significant at *p* < 0.05. Statistical significance of organ index, growth and carcass performance, and metabolites were determined by *t*-test. The statistical significance of α-diversity was performed with Wilcoxon tests.

## 3. Results

### 3.1. Improvement of growth and carcass traits in lambs fed a diet with alfalfa hay

To investigate the effects of *Leymus chinensis* hay and alfalfa hay on growth and carcass performance, we first compared IBW, FBW, ADG, carcass weight, and loin-eye area between the two groups. IBW was not significantly distinct, while total weight gain, FBW, and ADG were significantly greater in the Ms. group. Carcass weight, dressing percentage, and loin-eye area were also obviously increased in the Ms. group. However, lambs in the Ms. group had higher ADFI. The F/G was similar between the two groups (Table 1). In addition, we analyzed the organ index in lambs. Kidney index was increased in the Ms. group, while others including spleen, lung, liver, and heart index were not significantly affected (Table 2). These data indicated that compared to *Leymus chinensis* hay, alfalfa hay improved growth and carcass traits in lambs.

**Table 1:** Effects of *Leymus chinensis* hay and alfalfa hay on growth and carcass performance of sheep (*n* = 7).

Items	Lc group^f^ (average ± SE)	Ms group^g^ (average ± SE)	*p*-value^h^	
IBW^a^ (kg)	21.63 ± 1.58	22.43 ± 1.60	0.727	NS
FBW^b^ (kg)	30.71 ± 1.38	36.67 ± 1.76	0.020	^*^
ADG^c^ (kg)	0.14 ± 0.01	0.20 ± 0.00	0.009	^**^
ADFI^d^ (kg)	1.13 ± 0.04	1.32 ± 0.07	0.026	^*^
F/G^e^	10.71 ± 4.19	10.66 ± 2.46	0.639	NS
Carcass weight (kg)	13.29 ± 0.70	16.60 ± 1.18	0.033	^*^
Dressing percentage, %	43.21 ± 0.76	47.69 ± 0.58	0.0005	^***^
Loin-eye area (cm^2^)	15.39 ± 1.03	20.25 ± 1.35	0.014	^*^

a IBW, Initial body weight.

b FBW, Final body weight.

c ADG, Average daily gain.

d ADFI, Average daily feed intake.

e F/G, Feed to gain ratio.

f Lc group indicates lambs fed with *Leymus chinensis* hay.

g Ms group indicates lambs fed with alfalfa hay.

h ^*^p ≤ 0.05, ^**^p ≤ 0.01, ^***^p ≤ 0.001, NS indicates no significant differences.

**Table 2:** Effects of *Leymus chinensis* hay and alfalfa hay on organ index (*n* = 7).

Items	Lc group^a^ (average ± SE)	Ms group^b^ (average ± SE)	*p*-value^c^	
Spleen index, %	0.14 ± 0.01	0.15 ± 0.01	0.538	NS
Lung index, %	0.95 ± 0.04	1.07 ± 0.03	0.052	NS
Liver index, %	1.47 ± 0.06	1.63 ± 0.07	0.114	NS
Kidney index, %	0.27 ± 0.01	0.31 ± 0.00	0.001	^**^
Heart index, %	0.40 ± 0.02	0.43 ± 0.02	0.270	NS

a Lc group indicates lambs fed with *Leymus chinensis* hay.

b Ms group indicates lambs fed with alfalfa hay.

c ^*^p ≤ 0.05, ^**^p ≤ 0.01, NS indicates no significant differences.

### 3.2. Alterations of rumen bacterial diversity and composition between two groups

**Figure 1:**
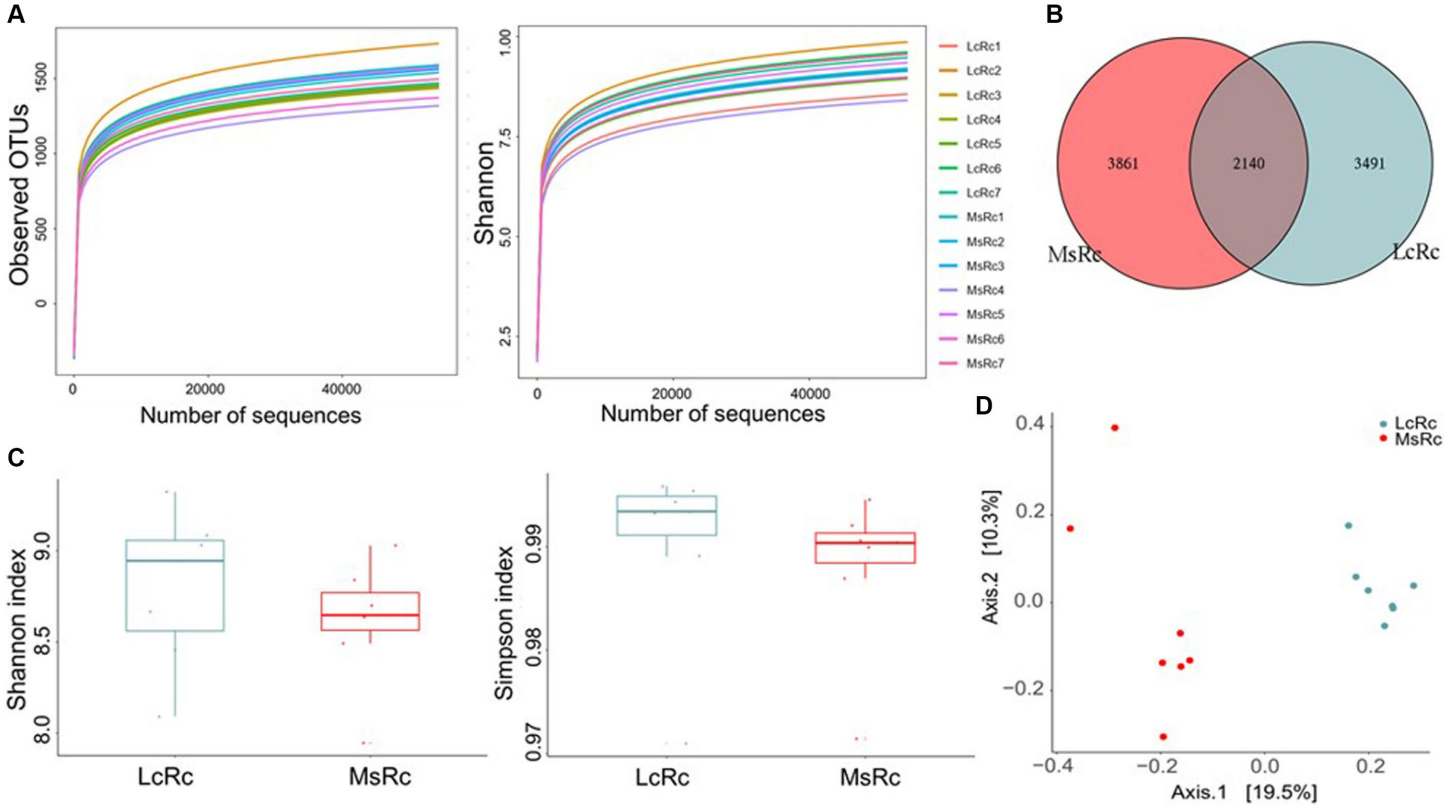
The diversity of the rumen microbiota of lambs fed a diet with *Lemus chinensis* hay and alfalfa hay. **(A)** Observed OTUs and Shannon curves of rumen microbiota. **(B)** The Venn diagram illustrates the overlap of microbial OTUs between the two groups. **(C)** The α-diversity includes Shannon and Simpson index. **(D)** The β-diversity presents as PCoA. *n* = 7 in each group. LcRc and MsRc indicate rumen contents from lambs fed with *Leymus chinensis* hay and alfalfa hay, respectively.

To investigate the effects of *Lemus chinensis* hay and alfalfa hay on rumen microbiota, we performed and analyzed bacterial 16S rDNA sequencing of rumen contents between two groups. The observed OTU and Shannon curves reached the saturation phase, suggesting the sufficient and reliable sequence depth captured in this study (Figure 1A). A total of 2,140 OTUs were identified in both groups, among which 3,491 and 3,861 specific OTUs were observed in the Lc and Ms. groups, respectively (Figure 1B). Shannon and Simpson indexes reflected the bacterial community richness and diversity. No significant differences were observed in the Shannon and Simpson index between the two groups (Figure 1C). The β-diversity presenting as Principal Co-ordinates Analysis (PCoA), Non-metric multidimensional scaling (NMDS), Principal Components Analysis (PCA), and Partial Least Squares Discrimination Analysis (PLS-DA) was further applied to analyze the variation of bacterial structure. The NMDS, PCA, and PLS-DA are in Supplementary Figure S3. The Lc group rumen contents (LcRc) formed a different rumen bacterial community clustered separately from the Ms group rumen contents (MsRc) (Figure 1D).

**Figure 2:**
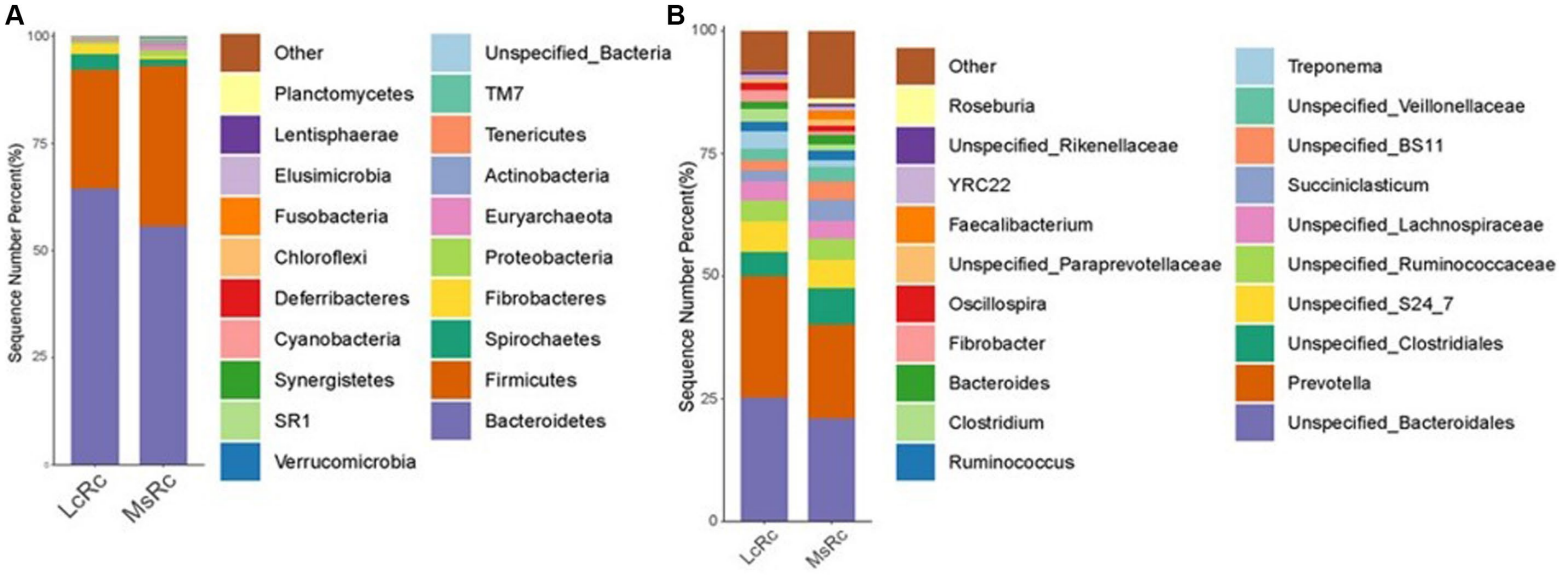
The composition of the rumen microbiota of lambs fed a diet with *Lemus chinensis* hay and alfalfa hay. Taxonomic distribution of rumen bacterial communities of different groups at phylum level **(A)** and genus level **(B)**. LcRc and MsRc indicate rumen contents from lambs fed with *Leymus chinensis* hay and alfalfa hay, respectively. “p_” denotes phylum-level classification, “f_” denotes phylum-level classification, and “g_” denotes genus-level classification.

To further define the composition of rumen microbiota in the LcRc and MsRc, the percentages of bacterial community abundance were analyzed at phylum and genus levels. At the phylum level, *Bacteroidetes*, *Firmicutes*, *Spirochaetes*, and *Fibrobacteres* were the dominant phyla (Figure 2A). The relative abundance of *Fibrobacteres*, *Bacteroidetes*, and *Spirochaetes* were obviously greater in the Lc group (Supplementary Table S2); the relative abundance of *Firmicutes*, *Actinobacteria*, *Fusobacteria*, *Verrucomicrobia*, and *Proteobacteria* were obviously greater in the MsRc (Supplementary Table S2). At the genus level, the top 20 genera were present with relatively high abundance (Figure 2B). The 17 genera significantly altered between two groups, among which only 5 genera, i.e., *Clostridiu*, *Fibrobacter*, *Selenomonas*, *BF311*, and *Treponema*, were greater in the LcRc. Most of the altered bacteria in the MsRc, including *Moryella*, *Blautia*, *p_75_a5*, *Faecalibacterium*, *Mogibacteriaceae*, *Anaerostipes*, *Bifidobacterium*, *Eggerthella*, *and Collinsella* belong to *Firmicutes* and *Actinobacteria* (Supplementary Table S3).

**Figure 3:**
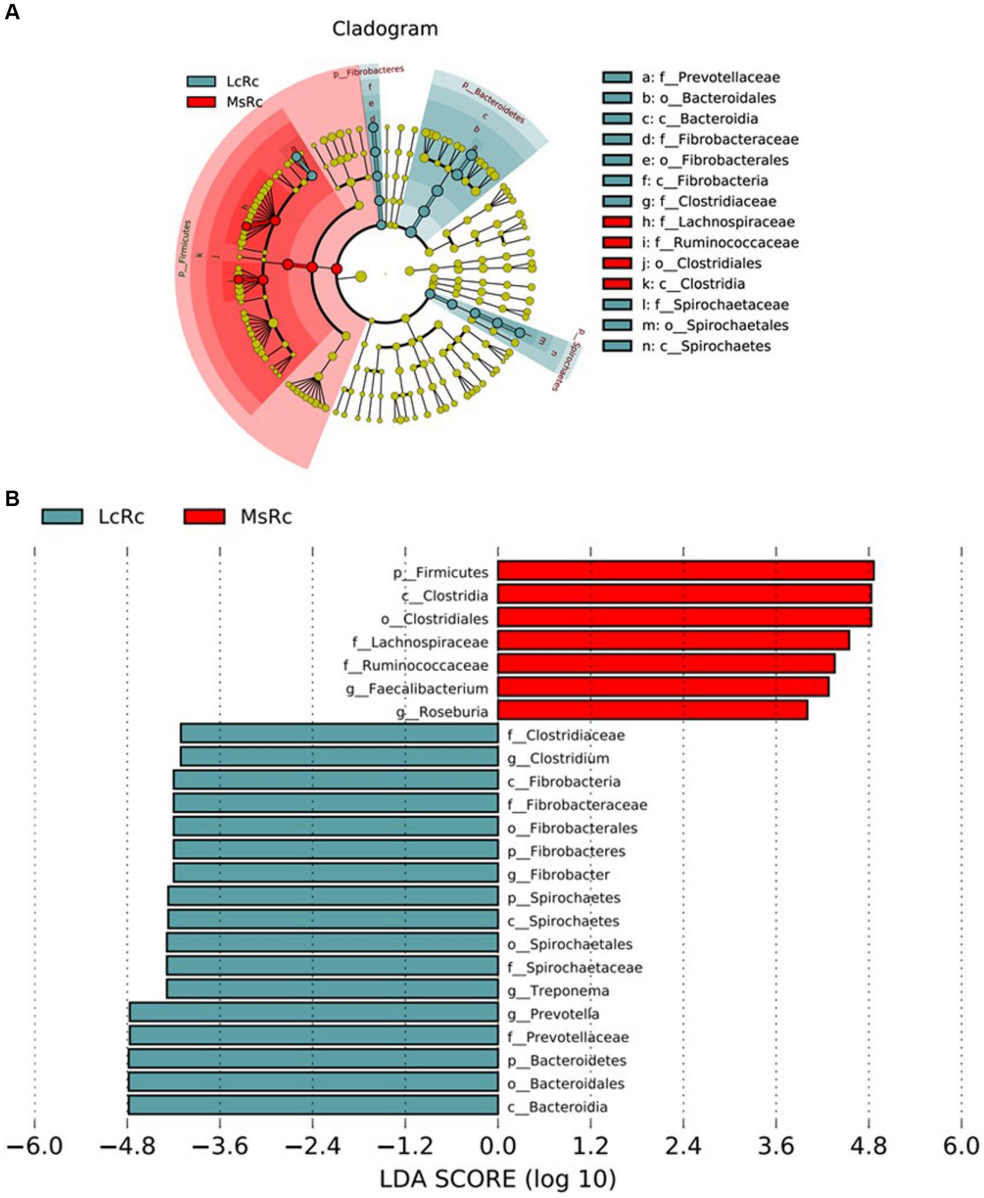
The effect of *Lemus chinensis* hay and alfalfa hay on the taxonomic diversity of the rumen bacteria. The cladogram **(A)** and histogram **(B)** show rumen bacterial taxa with a linear discriminant analysis (LDA) score >4.0 by LEfSe analysis. LcRc and MsRc indicate rumen contents from lambs fed with *Leymus chinensis* hay and alfalfa hay, respectively.

The rumen bacterial composition was further compared through LEfSe analysis with 4.0 as the threshold on the LDA score to identify specific species in each group. The constitution of rumen bacteria changed between LcRc and MsRc. A total of 17 taxa sequences were enriched in the LcRc and mainly belonged to *Fibrobacteres*, *Bacteroidetes*, and *Spirochaetes* (Figure 3). A total of 7 rumen bacteria mainly belonging to *Firmicute* were determined as enriched in the MsRc (Figure 3). Taken together, lambs in Lc and Ms. groups had distinct compositions of rumen bacterial community, which may lead to different growth performance, carcass performance, and metabolic profiles.

### 3.3. Key rumen microbiota associated with ADG, carcass weight, and body weight

**Figure 4:**
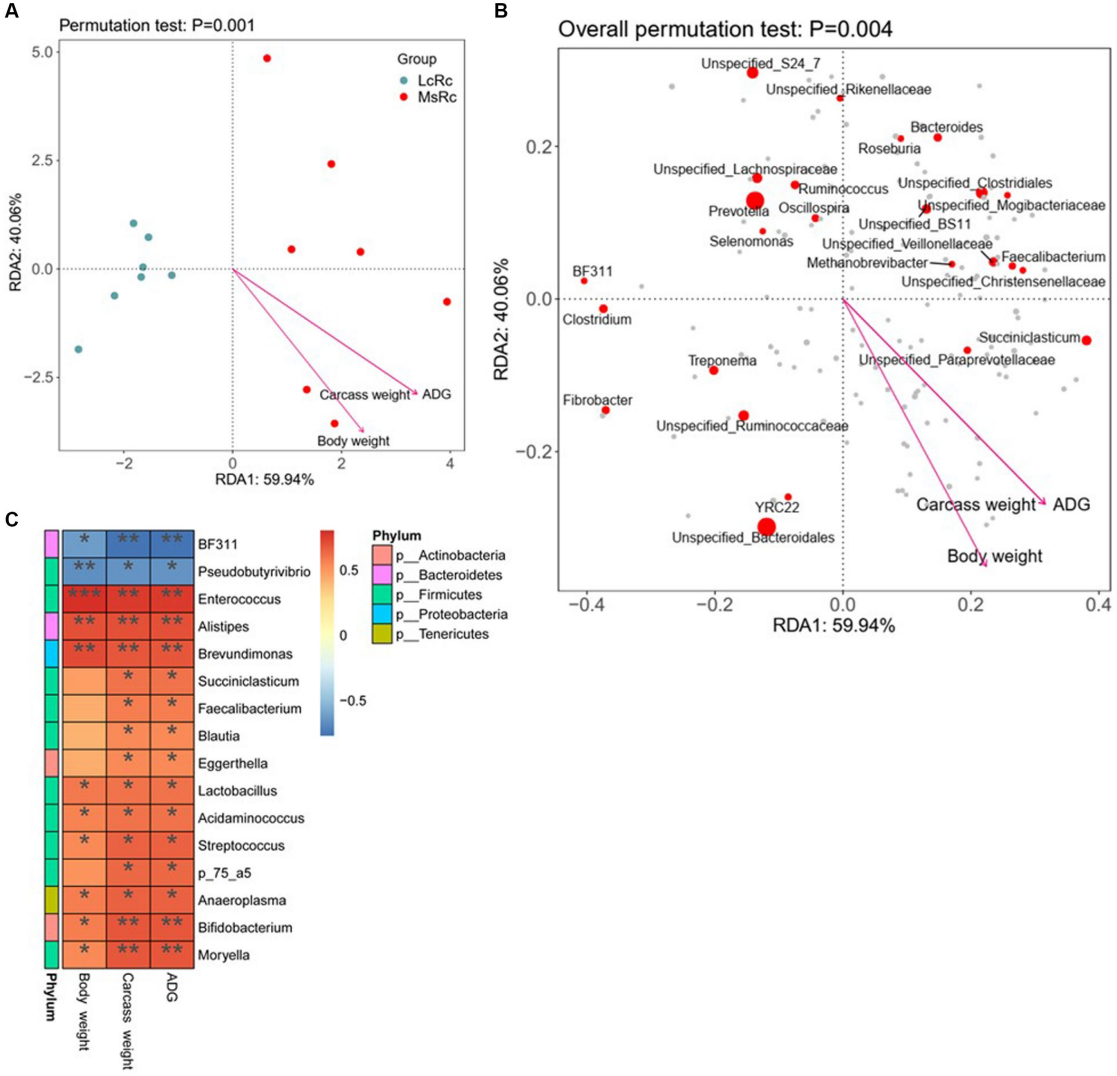
Relationship between key rumen bacteria with ADG, carcass weight, and body weight. **(A,B)** RDA was performed on genus-level taxonomic profile and host parameters including ADG, carcass weight, and body weight. Arrows indicate the correlation between community structure and host parameters. **(C)** Correlation analysis of key rumen microbiota with ADG, carcass weight, and body weight. Statistical significance was calculated by Spearman’s correlation analysis. ^*^p ≤ 0.05, ^**^p ≤ 0.01, ^***^p ≤ 0.001.

To further investigate the potential relationship between rumen microbiota variation and ADG, carcass weight, and body weight, we performed the RDA and Spearman’s correlation between differential rumen bacteria and host parameters. According to RDA, the *Lemus chinensis* hay and alfalfa hay treatments were distinct and two different clusters were observed. ADG, carcass weight, and body weight showed a significant correlation with bacterial community (Figures 4A,B). The Spearman’s correlation data showed that the relative abundance of *BF311* and *Pseudobutyrivibrio* were negatively related to ADG, carcass weight, and body weight (Figure 4C). Additionally, most genus-level bacteria such as *Enterococcus*, *Lactobacillus*, *Acidaminococcus*, and *Moryella* belonging to *Firmicutes* showed a strongly positive correlation with ADG, carcass weight, and body weight (Figures 4C). *Alistipes* belonging to *Bacteroidetes*, *Eggerthella* and *Bifidobacterium* belonging to *Bacteroidetes*, and *Anaeroplasma* belonging to *Tenericutes*, were also positively associated with ADG, carcass weight, and body weight (Figure 4C).

### 3.4. Metabolome profiles of longissimus dorsi

**Figure 5:**
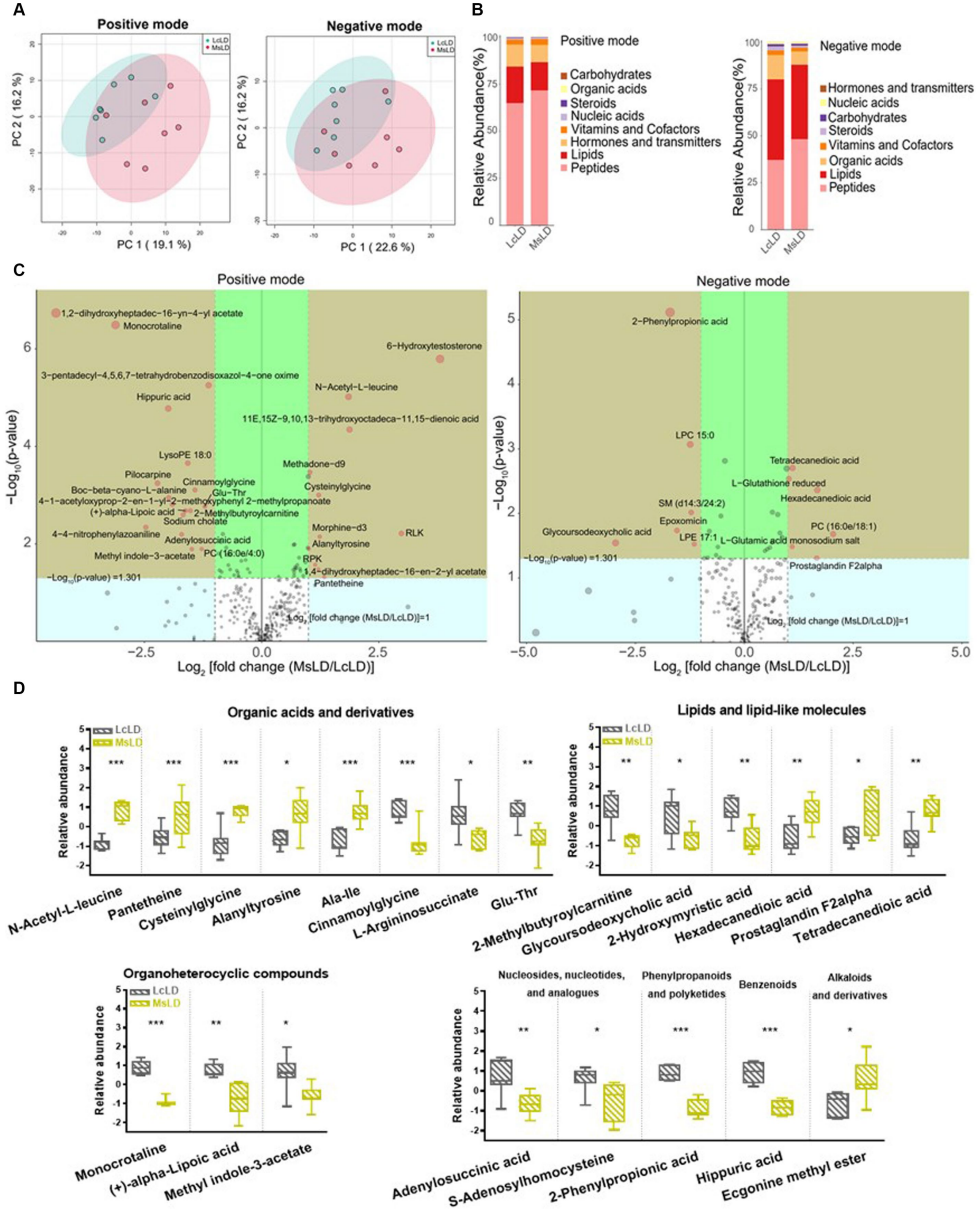
Effects of *Lemus chinensis* hay and alfalfa hay on metabolites of LD in lambs. **(A)** Scatter plots of the PCA model based on identified metabolite features. **(B)** Composition of metabolites in LcLD and MsLD. **(C)** Volcano plot of differential metabolites between LcLD and MsLD. **(D)** Boxplot of selected differential metabolites. *n* = 7 in each group. ^*^p ≤ 0.05, ^**^p ≤ 0.01, ^***^p ≤ 0.001. LcLD and MsLD indicate LD from lambs fed with *Leymus chinensis* hay and alfalfa hay, respectively.

**Figure 6:**
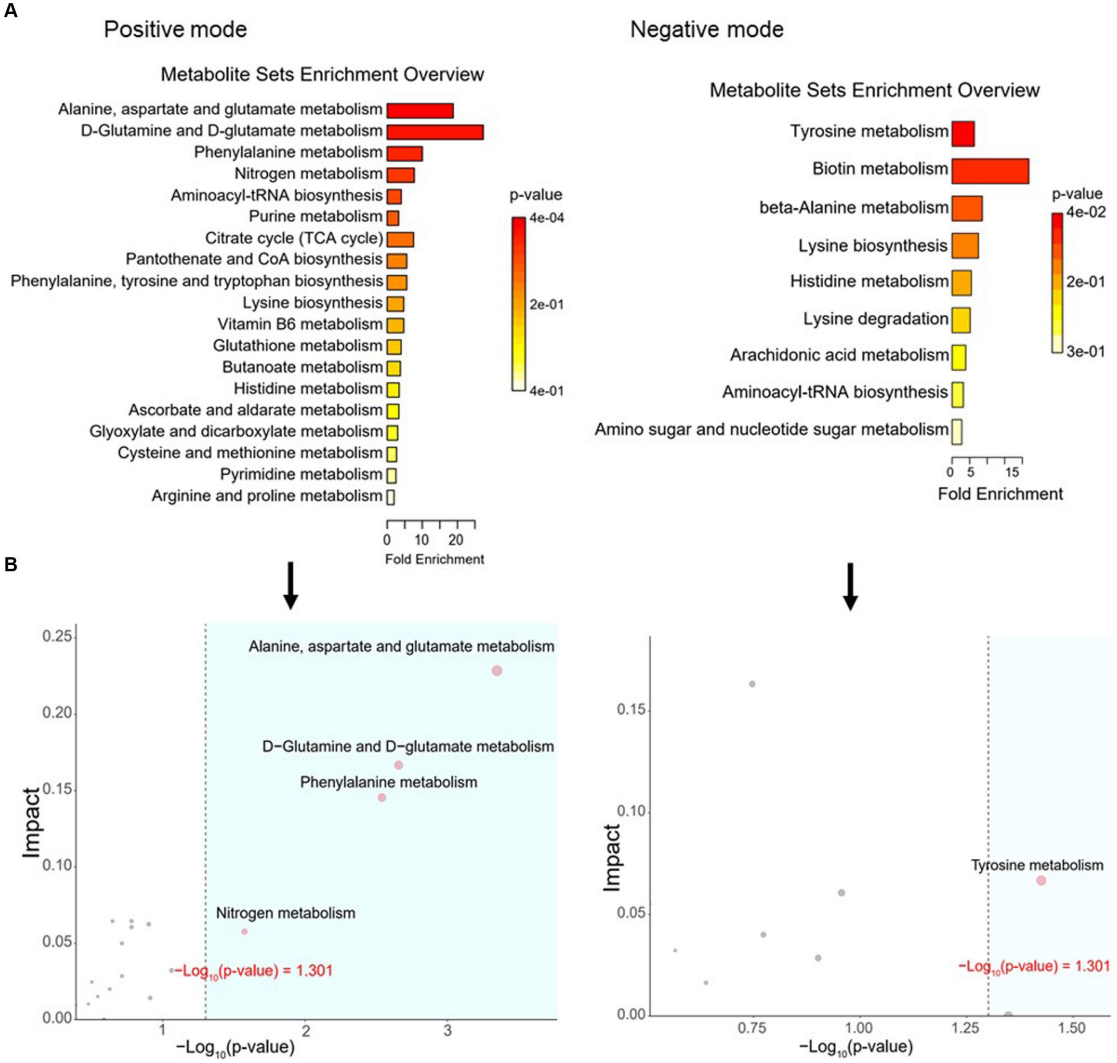
Pathway analysis of differential metabolites. **(A)** Enriched KEGG pathways of the comparison between LcLD and MsLD. **(B)** Pathway enrichment and topology analysis.

To define similarities and differences between lamb meat fed with *Leymus chinensis* hay and alfalfa hay, we performed and analyzed the untargeted metabolome profiles of LD samples. PCA score plots with positive and negative ionization modes revealed no significant clustering of the two treatment groups (Figure 5A), but further analysis revealed significant differences in metabolite composition. These metabolites mainly consisted of peptides, lipids, organic acids, steroids, carbohydrates, nucleic acids, vitamins and cofactors, hormones, and transmitters (Figure 5B). These two comparisons identified 96 significant differential metabolites (67 in the positive mode and 29 in the negative mode), among which 52 and 44 differential metabolites were elevated in the Lc and Ms. groups, respectively. In detail, organic acid and derivatives including N-Acetyl-L-leucine, Pantetheine, Cysteinyl glycine, Alanyl tyrosine, and Ala-Ile were greater in the Ms group longissimus dorsi (MsLD), while Cinnamoyl glycine, L-arginosuccinate, and Glu-Thr were greater in the Lc group longissimus dorsi (LcLD). Lipids and lipid-like molecules such as 2-methylbutyroylcarnitine, Glycoursodeoxycholic acid, and 2-Hydroxymyristic acid were higher in the LcLD, while Hexadecanediouc acid, prostaglandin F2alpha, and tetradecanedioic acid were higher in the MsLD. Interestingly, LcLD contained higher organoheterocyclic compounds including Momocrotaline, (+)-alpha-lipoic acid, Methyl indole-3-acetate, benzenoids (i.e., hippuric acid), phenylpropanoids, and polyketides (i.e., 2-phenylpropionic acid). MsLD had a higher relative abundance of alkaloids and derivates including ecgonine methyl ester (Figures 5C,D). Between *Leymus chinensis* hay and alfalfa hay treatment, peptides including Ala-Ile, Glu-Thr, N-Acetyl-Asp-Glu, gamma-glutamyl-cysteine, and gamma-glutamyl-L-leucine, significantly changed (Figures 5C,D). Furthermore, based on the pathway analysis, we found that these differential metabolites were mainly enriched in alanine, aspartate and glutamate metabolism, D-glutamine and D-glutamate metabolism, phenylalanine metabolism, nitrogen metabolism, and tyrosine metabolism (Figures 6A,B). Changed metabolites in these pathways were shown in Table 3, indicating that different forages affected amino acid composition and contents, and its metabolism, leading to various meat nutritional qualities.

**Table 3:** KEGG pathway enrichment of the changed metabolites for LcLD vs. MsLD.^a^

KEGG pathway	Changed metabolite	*p*-value	Upregulation
Alanine, aspartate, and glutamate metabolism	L-Glutamine	0.0004	LcLD
	2-Oxoglutaric acid		LcLD
	Adenylosuccinic acid		LcLD
D-Glutamine and D-glutamate metabolism	L-Glutamine	0.0022	LcLD
	2-Oxoglutaric acid		LcLD
Phenylalanine metabolism	L-Phenylalanine	0.0029	MsLD
	Phenylacetylglycine		MsLD
	Hippuric acid		LcLD
Nitrogen metabolism	L-Phenylalanine	0.0266	MsLD
	L-Glutamine		LcLD
Tyrosine metabolism	L-Dopa	0.0376	MsLD
	Homovanillic acid		MsLD
Biotin metabolism	L-Lysine	0.0449	MsLD

a LcLD and MsLD indicate LD from lambs fed with *Leymus chinensis* hay and alfalfa hay, respectively.

To facilitate the identification of potential biomarkers between the two groups, PLS-DA and OPLS-DA were applied to identify the major differences in metabolites. PLS-DA score plots showed the best separation of LcLD and MsLD samples (Supplementary Figure S2A). Several potential biomarkers with VIP >2 including Monocrotaline, Hippuric acid, 6-Hydroxytestosterone, N-Acetyl-L-leucine, LysoPE 18:0, Ala-Ile, Methadone-d9, Cysteinylglycine, Cinnamoylglycine, Pilocarpine, and 2-Phenylpropionic acid and were highlighted in the volcano plots (Supplementary Figure S2B). To minimize overfitting in the PLS-DA model, we further explored potential markers using OPLS-DA between groups. The analysis presented a cross-validated score plot (R2X = 0.311, R2Y = 0.993, Q2 = 0.737, *p*-value = 0.01) of the discriminating model between LcLD and MsLD in positive ionization mode. The cross-validated score plot (R2X = 0.454, R2Y = 0.995, Q2 = 0.526, *p*-value = 0.05) was shown in negative ionization mode. Identified biomarkers were the same as those identified by PLS-DA as having a trend toward being distinct between the two groups (Supplementary Figure S3). Additionally, random forest showed top differential metabolites including N-Acetyl-L-leucine, Hippuric acid, (+)-alpha-lipoic acid, and 2-Phenylpropionic acid (Supplementary Figure S4).

**Figure 7:**
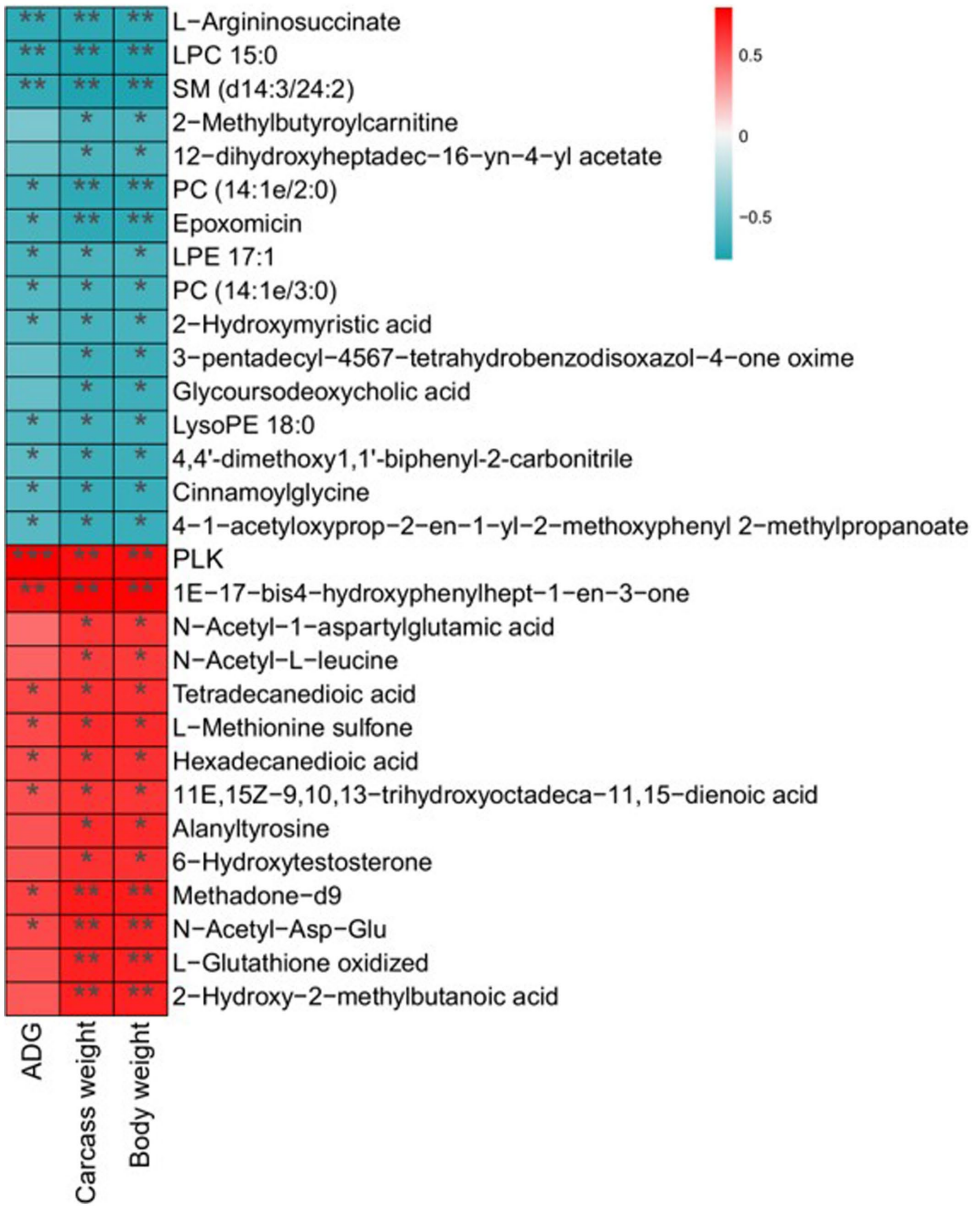
Correlation analysis of differential metabolites with ADG, carcass weight, and body weight. Statistical significance was calculated by Spearman’s correlation analysis. ^*^p ≤ 0.05, ^**^p ≤ 0.01, ^***^p ≤ 0.001.

Differential metabolites and potential biomarkers have been clarified as mentioned above. The correlation of potential biomarkers with growth performance was further analyzed. A total of 30 differential metabolites were significantly associated with ADG, carcass weight, and body weight. Among them, 14 metabolites including N-Acetyl-L-leucine, Methadone-d9, Hexadecanedioic acid, and alanyltyrosine were positively related to ADG, carcass weight, and body weight. Sixteen metabolites such as LysoPE 18:0, Cinnamoylglycine, and 2-Methylbutyroylcarnitine exhibited a negative relationship with growth performance (Figure 7).

### 3.5. Correlation of key rumen microbiota with differential metabolites

**Figure 8:**
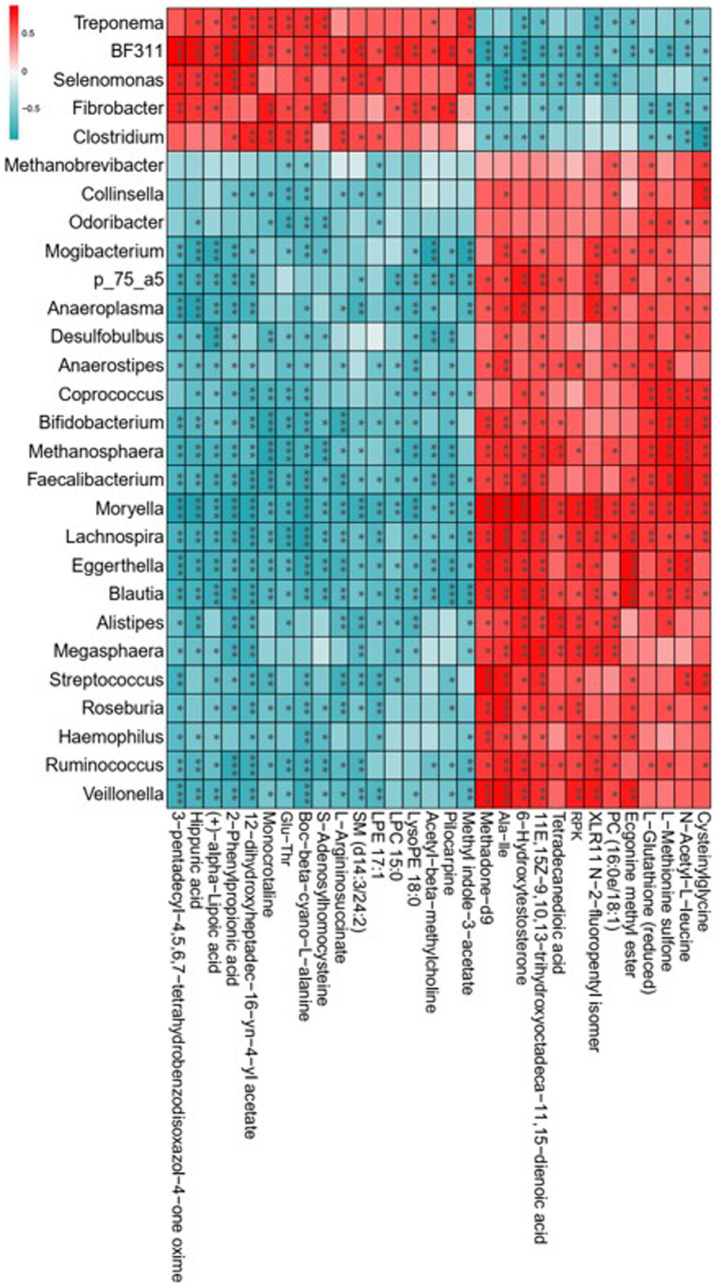
Correlation analysis of key rumen microbiota with differential metabolites. Statistical significance was calculated by Spearman’s correlation analysis. ^*^p ≤ 0.05, ^**^p ≤ 0.01, ^***^p ≤ 0.001.

The 16S rDNA sequencing revealed that lambs with the consumption of dietary *Leymus chinensis* hay and alfalfa hay exhibited distinct rumen microbiota communities. The metabolomics analysis indicated that exposure to *Leymus chinensis* hay and alfalfa hay resulted in different metabolic profiles of LD. Therefore, we explored the correlations between the rumen microbiota and the altered LD metabolites through Spearman’s correlation analysis, revealing high correlations between key rumen microbiota with differential metabolites (Figure 8). In detail, *Fibrobacter*, *Selenomonas*, *BF311*, *Treponema*, and *Clostridium* exhibited a strong positive relationship with organic acid and derivatives such as Glu-Thr, benzenoids including hippuric acid, phenylpropanoids and polyketides including 2-phenylpropionic acid, and organoheterocyclic compounds including Methyl indole-3-acetate, Momocrotaline, and (+)-alpha-lipoic acid. And they were negatively associated with other differential metabolites including Ala-Ile, N-Acetyl-L-leucine, Tetradecanedioic acid, and Methadone-d9. Other genera including *Bifidobacterium*, *Faecalibacterium*, *Blautia*, *Roseburia*, *Moryella*, *Streptococcus*, *[Ruminococcus]*, and *Odoribacter*, showed an obviously opposite correlation trend with the five genera mentioned above (Figure 8). These results indicated that the rumen bacterial compositions obviously and specifically impacted host LD metabolites.

## 4. Discussion

The forage is one of the main factor affecting ruminant production, rumen microbiota community, and meat quality. Previous studies explored ratios of various forages such as alfalfa hay, *Leymus chinensis* hay, *Vigna radiata* stalk, and corn stalk, which had a greater effect on the ruminants (5, 9, 12). To our knowledge, few studies have systematically compared the growth and carcass traits of lambs fed *Leymus chinensis* hay and alfalfa hay. In this study, we found that feeding with alfalfa hay increased lamb carcass weight, FBW, ADG, loin-eye area, and dressing percentage compared to those in the Lc group, supporting that those lambs fed with the alfalfa hay exhibited better carcass characteristics and growth performance.

The composition and diversity of rumen bacteria exhibit a close relationship with the host. Diet is one of the key factors that triggers changes in rumen microbial communities, alongside different environmental factors (33). To investigate the impacts of *Leymus chinensis* hay and alfalfa hay on the host rumen bacterial community, 16S rDNA amplicon sequencing was performed and analyzed. Obviously, there was no significant difference in Shannon and Simpson indexes in the LcRc and MsRc. This indicates no significant altered in the diversity of rumen bacteria.

Exploring changes in rumen microbiota, which are closely related to muscle metabolites, can help to investigate the mechanisms for improving lamb meat quality under different roughage conditions. At the phylum level, *Firmicutes* and *Bacteroidetes* were the major bacterial phyla in the rumen as reported (20, 22), whose relative abundances were significantly distinct in the LcRc and MsRc. *Firmicutes* are dominant across rumen bacterial communities and consist of various fibrolytic and cellulolytic bacterial genera (34). *Bacteroidetes* are beneficial for digesting carbohydrates (35). Then, we analyzed differences in key rumen bacteria at the genus level between the two groups and their correlation with metabolite deposition in the LD. *Unspecified bacteroidales* and *Prevotella* were the dominant bacterial genera in both groups. Based on the LEfSe analysis, lambs in the Lc group have a higher relative abundance of *Prevotella*, which plays an important role in polysaccharide and protein metabolism, and utilization of hemicelluloses (20). *Fibrobacter* was also higher in the LcRc, which is a cellulolytic bacteria in the rumen of ruminants (36). This may be related to the higher fiber intake of lambs in the Lc group, while we found that they positively regulate Glu-Thr levels in the LD. Dipeptides are important flavor precursors in lamb meat (37), suggesting that rumen microorganisms may influence lamb meat quality by regulating flavor substances in muscle after feeding *Leymus chinensis* hay. It has been reported that *Fibrobacter* was able to synthesize oligosaccharides and contributed to rapidly adapting to sudden environmental changes (33). In addition, three genera mainly belonging to the fibrolytic bacteria, such as *Selenomonas*, *BF311*, and *Treponema* were obviously elevated in the LcRc. *Treponema* is closely related to pectin treatments due to its ability to degrade pectin, which shares mutual interaction with *Fibrobacter* (33, 38). *Selenomonas* can oxidize lactate and produce propionic acid as a main fermentation product (39). And the correlation between these three fibrolytic bacteria and the metabolite levels of the LD had the same trend as *Fibrobacter*, suggesting that they may have the same mechanism of action. Most of the significant differential bacteria, such as *Bifidobacterium*, *Faecalibacterium*, *Mogibacterium*, *Anaerostipes*, *Alistipes*, and *Blautia*, were elevated in the MsRc. A previous study has shown that *Bifidobacterium* is saccharolytic bacteria can generate acetate and lactate (10). *Faecalibacterium* is one of the butyrate-producing bacteria linked to higher weight gain, and displays anti-inflammatory action (40). *Anaerostipes* is also a butyrate-producing bacterium (41). In the present study also the relative abundance of *Faecalibacterium* was found to be positively correlated with body weight and carcass weight. Changes in *Mogibacterium* are associated with feed efficiency in ruminants (42). Additionally, *Blautia* has been proven to enhance beneficial anti-inflammatory effects of hosts (43). These results suggested that alfalfa hay may have altered feed efficiency, influenced volatile fatty acid production, and also facilitated rumen resistance to inflammation. Taken together, the forage types, i.e., *Leymus chinensis* hay and alfalfa hay, resulted in different rumen bacterial compositions of lambs.

The compositions of nutrient metabolites have a direct impact on meat quality (44). Thus, meat metabolic profiles aroused great public concern. Amino acids are important ingredients for the nutritional value of mutton (45). According to the analysis of untargeted metabolomics, we found significant differences in amino acid-related metabolites in meat between Lc and Ms. groups. Some amino acid metabolism, including alanine, aspartate, and glutamate metabolism, phenylalanine metabolism, D-glutamine, and D-glutamate metabolism, and tyrosine metabolism were changed, suggesting forages regulated amino acid contents and metabolism, and thus affected the nutritional quality of meat. Free amino acids and dipeptides are important flavor precursors in meat (37). Importantly, some peptides have bioactive properties (37). Cysteinylglycine belonging to the dipeptide is a structural component of glutathione (46), which was higher in the MsLD. Between *Leymus chinensis* hay and alfalfa hay treatment, peptides including Ala-Ile, Glu-Thr, N-Acetyl-Asp-Glu, gamma-glutamyl-cysteine and gamma-glutamyl-L-leucine changed significantly. The gamma-glutamyl peptide, gamma-glutamyl-L-leucine, has been recognized as a kokumi-imparting molecule (47). Another gamma-glutamyl peptide, gamma-glutamyl-cysteine, can activate the calcium-sensing receptor and then impart the kokumi peptide-induced responses (48).

Rumen microorganisms provide energy for muscle metabolism to the host mainly in the form of volatile fatty acids (49). Feeding *Leymus chinensis* hay and alfalfa hay not only altered the substrates available for rumen fermentation but may have also altered the distribution of various fatty acids in the rumen, thereby affecting the composition of the rumen microbiota and the levels of LD muscle metabolites (50). For example, Du et al. (51) found that *Ruminiclostridium_6* and *U29-B03* might be participating in carbohydrate metabolism to produce volatile fatty acids, which promote IMF deposition affecting tenderness in muscles. The significant changes in the relative abundance of the associated bacteria in the Lc and Ms. groups in the present study may similarly cause alterations in the levels of metabolites in muscle via the volatile fatty acid pathway. However, more research would be required to reveal the connection between different roughages and changes in rumen microbiota and muscle metabolites.

## 5. Conclusion

Compared to lamb with the *Leymus chinensis* hay diet, lambs fed with alfalfa hay exhibited better growth performance and carcass performance. *Leymus chinensis* hay led to the enrichment of the genera *Fibrobacter*, *Treponema*, *Selenomonas*, and *BF311*. Alfalfa hay led to the enrichment of *Blautia*, *Anaerostipes*, *Faecelibacterium*, *Alistipes*, *Bifidobacterium*, and other genera. Ruminal bacteria after feeding different roughages affect the quality of lamb meat by influencing the metabolism of several amino acids and polypeptides in lamb meat. It was also demonstrated that alanine, aspartate and glutamate metabolism, D-glutamine and D-glutamate metabolism, phenylalanine metabolism, nitrogen metabolism, and tyrosine metabolism were the key metabolic pathways involved after feeding *Leymus chinensis* hay and alfalfa hay. In addition, conjoint analysis of rumen microbes and metabolomics indicated a close relationship between rumen microbial composition and muscle metabolites. These results have important significance for the future adequate and rational utilization of pasture to improve the quality of lamb meat.

## Data availability statement

The datasets presented in this study can be found in online repositories. The names of the repository/repositories and accession number(s) can be found at: https://www.ncbi.nlm.nih.gov/bioproject; PRJNA995904.

## Ethics statement

The animal study was approved by Animal Care and Use Committee of Inner Mongolia University (Approval No. IMU-sheep-2020-041). The study was conducted in accordance with the local legislation and institutional requirements.

## Author contributions

HW: Writing – original draft. LiM: Data curation, Writing – review & editing. LaM: Writing – review & editing, Data curation.
