# Supplementary material for: Effects of Leymus chinensis hay and alfalfa hay on growth performance, rumen microbiota, and untargeted metabolomics of meat in lambs
Source: Front Vet Sci. 2023 Nov 16;10:1256903. doi: 10.3389/fvets.2023.1256903 (PMC10687458; doi:10.3389/fvets.2023.1256903)
Supplement: Supplementary file 1 [file Data_Sheet_1.docx]

Supplementary Material

# Supplementary Tables

**Table S1** Dietary concentrate components and chemical composition

| **Item** | **Lc group** | **Ms group** |
| --- | --- | --- |
| *Dietary concentrate components* |  |  |
| Corn | 62 | 62 |
| Wheat bran | 12 | 12 |
| Soybean meal | 8 | 8 |
| Cottonseed meal | 12 | 12 |
| NaHCO_3_ | 1 | 1 |
| Salt | 1 | 1 |
| Premix^1^ | 4 | 4 |
| *Roughage* |  |  |
| *Leymus chinensis* hay | 71.19 | - |
| Alfalfa hay | - | 75.19 |
| *Chemical composition* |  |  |
| Dry matter (DM) | 94.82 | 94.33 |
| Crude protein, % of DM | 10.17 | 16.54 |
| Crude ash, % of DM | 7.61 | 8.35 |
| Crude fat, % of DM | 1.12 | 0.58 |
| Neutral detergent fiber, % of DM | 40.83 | 34.78 |
| Acid detergent fiber, % of DM | 39.65 | 33.59 |
| Total Calcium, % of DM | 0.66 | 1.06 |
| Total Phosphorus, % of DM | 0.21 | 0.21 |

^1^ Per kg of premix contained VA 280000 IU, VD_3_ 100000 IU, VE 280 IU, Fe 7 g, Cu 625 mg, Zn 3.75 g, Mn 3 g, I 80 mg, Se 10 mg, Co 35 mg.

**Table S2** Relative abundance of microbiota at the phylum level between Lc group and Ms group

| Taxonomy | LcRc: Mean±SE^1^ % | MsRc: Mean±SE % | P-Value |
| --- | --- | --- | --- |
| *Proteobacteria* | 0.58±0.064 | 1.37±0.091 | 0.0000 |
| *Spirochaetes* | 3.64±0.54 | 1.58±0.28 | 0.0082 |
| *Firmicutes¬* | 27.71±1.84 | 37.44±2.47 | 0.0090 |
| *Bacteroidetes* | 64.45±1.92 | 55.79±2.34 | 0.0148 |
| *Fibrobacteres* | 2.37±0.53 | 0.75±0.15 | 0.0226 |
| *Fusobacteria* | 0.00074±0.00074 | 0.018±0.0069 | 0.0428 |
| *Verrucomicrobia* | 0.071±0.0090 | 0.17±0.040 | 0.0451 |
| *Euryarchaeota* | 0.24±0.11 | 1.04±0.33 | 0.0557 |
| *Elusimicrobia* | 0.0038±0.0019 | 0.0085±0.0019 | 0.1050 |
| *SR1* | 0.15±0.076 | 0.027±0.0061 | 0.1547 |
| *Tenericutes* | 0.38±0.056 | 0.91±0.35 | 0.1873 |
| *Synergistetes* | 0.073±0.0096 | 0.10±0.021 | 0.2311 |
| *TM7* | 0.059±0.017 | 0.41±0.34 | 0.3381 |
| *Acidobacteria* | 0.00053±0.00053 | 0.00±0.00 | 0.3559 |
| *Chlorobi* | 0.00043±0.00043 | 0.00±0.00 | 0.3559 |
| *LD1* | 0.00039±0.00039 | 0.00±0.00 | 0.3559 |
| *Unspecified_Bacteria* | 0.19±0.040 | 0.26±0.070 | 0.3919 |
| *Cyanobacteria* | 0.048±0.015 | 0.066±0.021 | 0.5160 |
| *Chloroflexi* | 0.0070±0.0035 | 0.012±0.0071 | 0.5381 |
| *Lentisphaerae* | 0.0022±0.0017 | 0.0045±0.0032 | 0.5463 |
| *Planctomycetes* | 0.0015±0.00069 | 0.0023±0.0023 | 0.7283 |

^1^ SE, standard error.

^2^ P-Value less than 0.05 were considered significantly different.

**Table S3** Relative abundance of significantly difference microbiota at the genus level between the Lc group and Ms group

| Taxonomy | LcRc: Mean±SE^1^ % | MsRc: Mean±SE % | P-Value |
| --- | --- | --- | --- |
| *BF311* | 0.83±0.064 | 0.17±0.023 | <0.001 |
| *Treponema* | 3.54±0.55 | 1.36±0.28 | <0.01 |
| *Selenomonas* | 0.58±0.085 | 0.31±0.088 | <0.05 |
| *Fibrobacter* | 2.36±0.53 | 0.75±0.15 | <0.05 |
| *Clostridium* | 2.79±0.38 | 1.43±0.20 | <0.05 |
| *Moryella* | 0.00±0.00 | 0.039±0.0093 | <0.01 |
| *Blautia* | 0.025±0.0070 | 0.60±0.13 | <0.01 |
| *p_75_a5* | 0.0085±0.0040 | 0.039±0.0096 | <0.05 |
| *Faecalibacterium* | 0.015±0.011 | 1.89±0.50 | <0.01 |
| *Mogibacterium* | 0.0061±0.0032 | 0.025±0.0059 | <0.05 |
| *Anaerostipes* | 0.012±0.0054 | 0.092±0.023 | <0.05 |
| *Bifidobacterium* | 0.023±0.015 | 0.28±0.070 | <0.01 |
| *Collinsella* | 0.011±0.011 | 0.093±0.032 | <0.05 |
| *Eggerthella* | 0.00±0.00 | 0.019±0.0057 | <0.05 |
| *Desulfobulbus* | 0.0051±0.0027 | 0.026±0.0059 | <0.05 |
| *Methanosphaera* | 0.00039±0.00039 | 0.048±0.011 | <0.01 |
| *Alistipes* | 0.00±0.00 | 0.024±0.0097 | <0.05 |

^1^ SE, standard error.

# Supplementary Figures

#
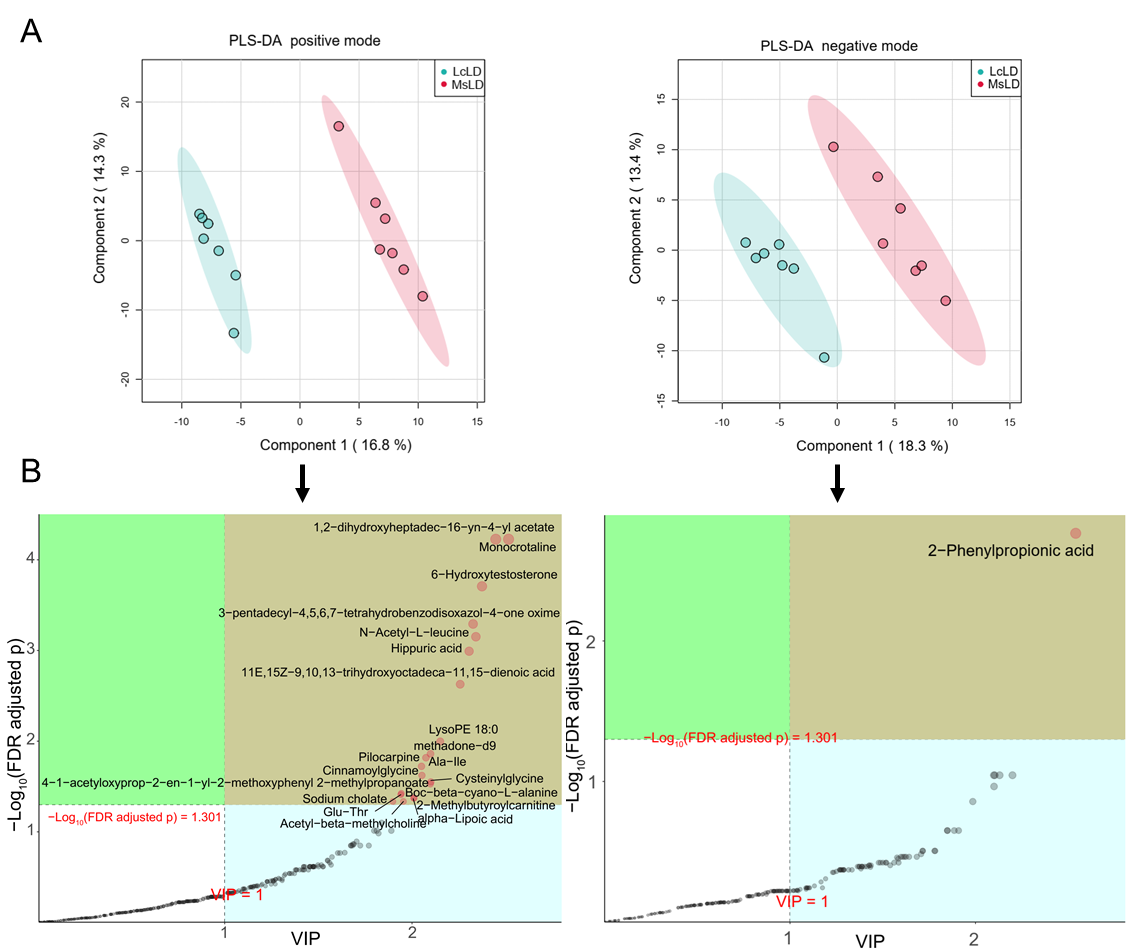


# Supplementary Figure 1. Potential biomarkers analysis using Partial Least Square-Discriminant Analysis (PLS-DA). PLS-DA score plots (A) and volcano plots (B) of all samples. VIP is short for Value Importance in Projection. LcLD and MsLD indicate LD from lambs fed with *Leymus chinensis* hay and alfalfa hay, respectively.


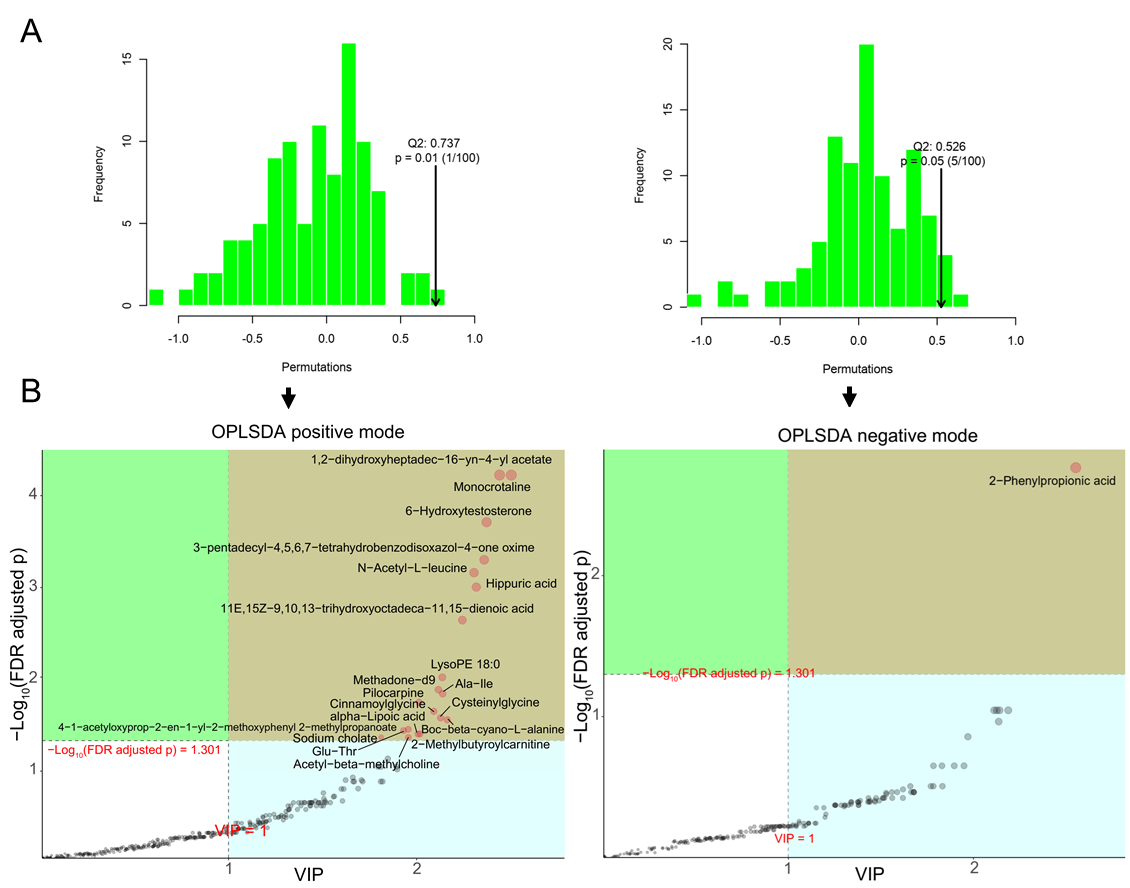


# Supplementary Figure 2. Potential biomarkers analysis using Orthogonal Partial Least Squares Discrimination Analysis (OPLS-DA). Permutation test (A) and volcano plots (B) of all samples. VIP is short for Value Importance in Projection. LcLD and MsLD indicate LD from lambs fed with *Leymus chinensis* hay and alfalfa hay, respectively.


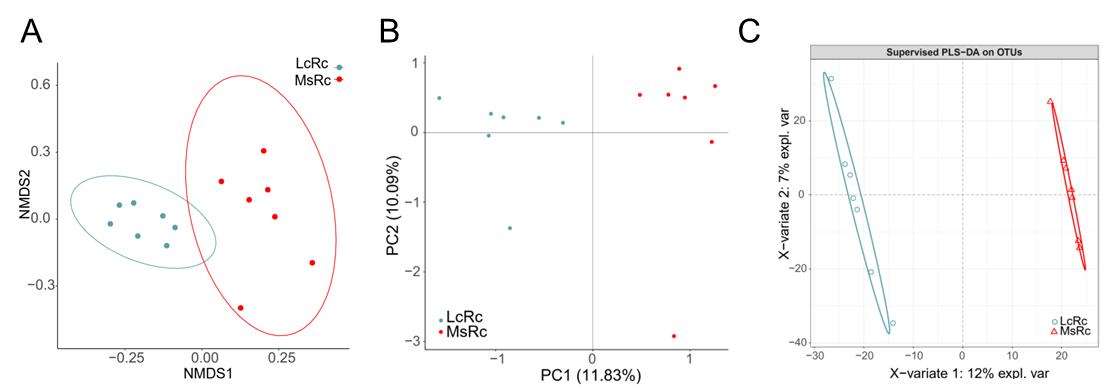


# Supplementary Figure 3. The β-diversity presenting as NMDS (A), PCA (B), and PLS-DA (C). LcRc and MsRc indicate rumen fluids from lambs fed with *Leymus chinensis* hay and alfalfa hay, respectively.


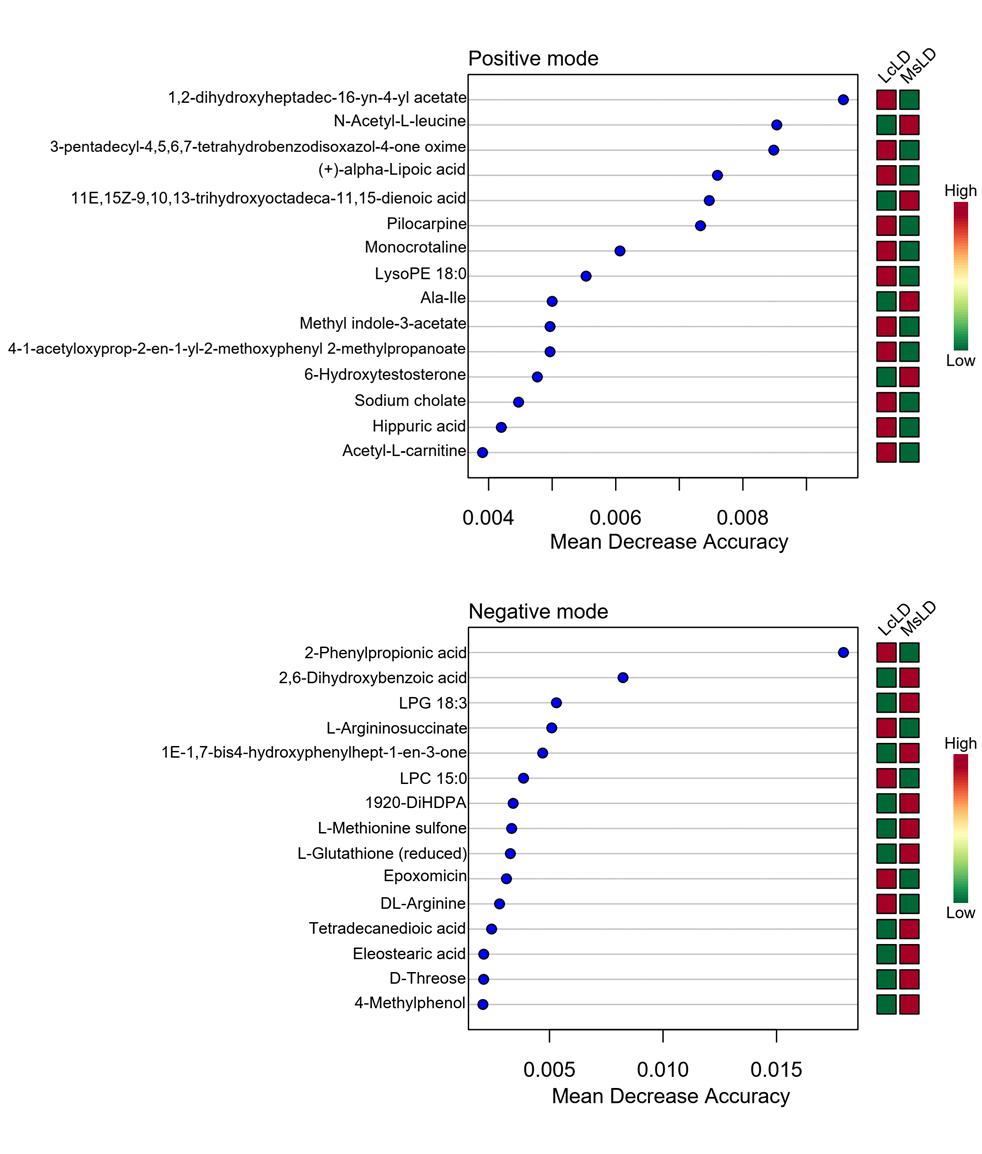


**Supplementary Figure 4.** Random Forest showing top metabolites between the two groups. LcLD and MsLD indicate LD from lambs fed with *Leymus chinensis* hay and alfalfa hay, respectively.
